# Discrimination of speech and non-speech sounds following theta-burst stimulation of the motor cortex

**DOI:** 10.3389/fpsyg.2014.00754

**Published:** 2014-07-15

**Authors:** Jack C. Rogers, Riikka Möttönen, Rowan Boyles, Kate E. Watkins

**Affiliations:** ^1^Department of Experimental Psychology, University of OxfordOxford, UK; ^2^School of Psychology, University of BirminghamBirmingham, UK

**Keywords:** continuous theta-burst stimulation (cTBS), transcranial magnetic stimulation (TMS), primary motor cortex, auditory discrimination, sensorimotor, categorical perception

## Abstract

Perceiving speech engages parts of the motor system involved in speech production. The role of the motor cortex in speech perception has been demonstrated using low-frequency repetitive transcranial magnetic stimulation (rTMS) to suppress motor excitability in the lip representation and disrupt discrimination of lip-articulated speech sounds (Möttönen and Watkins, 2009). Another form of rTMS, continuous theta-burst stimulation (cTBS), can produce longer-lasting disruptive effects following a brief train of stimulation. We investigated the effects of cTBS on motor excitability and discrimination of speech and non-speech sounds. cTBS was applied for 40 s over either the hand or the lip representation of motor cortex. Motor-evoked potentials recorded from the lip and hand muscles in response to single pulses of TMS revealed no measurable change in motor excitability due to cTBS. This failure to replicate previous findings may reflect the unreliability of measurements of motor excitability related to inter-individual variability. We also measured the effects of cTBS on a listener’s ability to discriminate: (1) lip-articulated speech sounds from sounds not articulated by the lips (“ba” vs. “da”); (2) two speech sounds not articulated by the lips (“ga” vs. “da”); and (3) non-speech sounds produced by the hands (“claps” vs. “clicks”). Discrimination of lip-articulated speech sounds was impaired between 20 and 35 min after cTBS over the lip motor representation. Specifically, discrimination of across-category ba–da sounds presented with an 800-ms inter-stimulus interval was reduced to chance level performance. This effect was absent for speech sounds that do not require the lips for articulation and non-speech sounds. Stimulation over the hand motor representation did not affect discrimination of speech or non-speech sounds. These findings show that stimulation of the lip motor representation disrupts discrimination of speech sounds in an articulatory feature-specific way.

## INTRODUCTION

Our ability to categorize acoustic speech signals is integral to accurate speech perception. Rather than perceiving continuous variations in speech in a linear fashion, variations along an acoustic continuum tend to be perceived categorically. A hallmark of categorical perception is that listeners are better at discriminating two sounds from opposite sides of the phonetic category boundary compared to two sounds with an equivalent acoustic distance that fall on the same side of the category boundary (Liberman et al., 1957; Repp, 1984). According to Liberman’s motor theory of speech perception (Liberman et al., 1967; Liberman and Mattingly, 1985) the listener perceives speech by simulating the “intended articulatory gestures” of the speaker and this affects the ability to categorize speech sounds.

This proposed link between speech perception and production remains a topic of active investigation and debate (e.g., Scott et al., 2009; Pulvermüller and Fadiga, 2010; Hickok et al., 2011). A series of studies have shown that listening to speech activates parts of the premotor and primary motor (M1) cortex in the brain that are important for speech production (e.g., Fadiga et al., 2002; Watkins et al., 2003; Wilson et al., 2004; Pulvermüller et al., 2006; Roy et al., 2008; Murakami et al., 2011). Functional imaging activity is observed in the lip and tongue representations in M1 during listening to speech sounds produced using the lip and tongue articulators (e.g., the phonemes /p/ and /t/), respectively (Pulvermüller et al., 2006). Studies using single pulses of transcranial magnetic stimulation (TMS) over the lip representation of the left M1 to elicit motor-evoked potentials (MEPs) in the lip muscles found that listening to speech enhanced motor excitability (Watkins et al., 2003; Murakami et al., 2011). Similarly, MEPs recorded from the tongue in response to single-pulse TMS showed facilitation specifically when participants listened to words that included speech sounds produced by the tongue (Fadiga et al., 2002).

Despite this growing body of evidence, the functional role of motor representations of articulators in speech perception remains unclear. Brain imaging and single-pulse TMS studies that demonstrate increased activity or excitability of motor areas during speech perception cannot answer key questions about whether these changes contribute to speech perception or are merely correlates of it. It is possible to examine whether these regions contribute to speech perception by using repetitive TMS (rTMS) to temporarily disrupt activity in the motor cortex. Interfering with the function of a specific cortical area (i.e., using TMS to create a “virtual lesion”) allows exploration of causal relationships between the stimulated brain region and behavioral performance (see Devlin and Watkins, 2007; Möttönen et al., 2014). Several previous studies using such methods demonstrated the contribution of the left premotor or primary motor cortex (M1) to performance in speech perception tasks (e.g., Meister et al., 2007; D’Ausilio et al., 2009; Sato et al., 2009; Bartoli et al., 2013). For instance, low-frequency rTMS has been shown to suppress motor excitability of the lip representation in left M1 temporarily (e.g., Möttönen and Watkins, 2009). This TMS-induced disruption of the motor lip representation also impaired the ability of listeners to categorically perceive and discriminate speech sounds drawn from acoustic continua ranging between lip- and tongue-articulated phonemes (e.g., “ba” vs. “da” and “pa” vs. “ta”; Möttönen and Watkins, 2009). The disruption did not impair the ability to categorically perceive or discriminate sounds from acoustic continua that are not articulated by the lips (e.g., “ka” vs. “ga” and “da” vs. “ga”). The effect was also specific to the site of stimulation, since disruption of the hand representation within left M1 had no effect on behavioral performance. These findings suggest that the motor representation of the articulators in left M1 contributes to discrimination of speech sounds in an articulator-specific way.

One methodological limitation of low-frequency rTMS, however, is that the duration of the observed modulatory effect is roughly equivalent to the length of stimulation (i.e., the effects last approximately 15 min following 15 min of rTMS). Another form of rTMS, continuous theta-burst stimulation (cTBS), has been shown to produce long-lasting (e.g., 60 min) suppression of motor excitability following only a short train (e.g., 40 s) of stimulation with maximum effects occurring between 20 and 40 min after cTBS (Huang et al., 2005). During cTBS, low-intensity bursts of high-frequency (50 Hz) rTMS are repeated at 5 Hz (i.e., the theta-frequency). Even though adverse effects attributed to theta-burst stimulation (TBS) are reported to be extremely mild and infrequent (e.g., Grossheinrich et al., 2009; Oberman et al., 2011), safety guidelines regarding the use of TBS have yet to be published. A degree of caution in its application is advised, therefore. Here, we used cTBS to stimulate the motor representation of the lips in M1, which allowed us a longer window of time during which we could test auditory discrimination abilities for a wider range of stimuli than tested in previous studies.

In the current study, we delivered 40 s of cTBS over the lip or hand representation of left M1. We assessed changes in cortical excitability within each target region over time by recording MEPs from the lips and hand before and after cTBS. The main aim of the experiment was to replicate and extend our previous findings using low frequency rTMS (Möttönen and Watkins, 2009), by assessing whether cTBS over the lip representation in M1 also impairs discrimination of speech sounds that require the lips for articulation. It has been suggested that rTMS-induced impairments in behavioral performance observed previously in the context of a same-different paradigm (e.g., Möttönen and Watkins, 2009) may reflect changes in response bias rather than perceptual processes important for speech (Hickok, 2010). A potential disadvantage of the same–different paradigm is that listeners may favor one of the response alternatives, resulting in a subjective bias towards “same” or “different” responses (Gerrits and Schouten, 2004; Macmillan and Creelman, 2005). We aimed to avoid this potential confound by using a variant of the ABX-discrimination task, AXB, the prototypical discrimination test used for assessing categorical perception. In an AXB-type task, the second stimulus (X) is identical to either the first (A) or the third (B) stimulus. All stimuli in this study were presented at two different inter-stimulus intervals (ISIs; 200 and 800 ms). Previous studies have shown that variations are retained in acoustic short-term memory if a short ISI (200–300 ms) is used (Pisoni and Lazarus, 1974; Pisoni and Tash, 1974; Pisoni, 1977; Massaro and Cohen, 1983). If the ISI exceeds the life span of auditory memory then an abstract, phonetic label based on pre-established categories is used to discriminate speech sounds (Massaro and Cohen, 1983; Gerrits and Schouten, 2004). Manipulating the ISI between sounds provided an opportunity to assess potential differences in discrimination strategy related to auditory memory versus phonetic-categorization. An impairment in discrimination resulting from TMS over the lip representation in M1, particularly at the longer ISI (800 ms), would also be consistent with findings from previous TMS studies demonstrating a role for the motor system in phonological segmentation and verbal working memory processes (Romero et al., 2006; Sato et al., 2009). The current study also differed further from our previous work in that the stimuli included recordings of natural speech sounds from which high-quality place-of-articulation continua were generated using a channel-vocoder (“Straight”; Kawahara et al., 1999). The continua ranged from lip- to tongue-articulated phonemes (“ba”–“da”) and phonemes that do not involve the lips in their articulation (“da”–“ga”). In addition to speech sounds, we also aimed to determine whether cTBS-induced disruption of the hand motor representation affected discrimination of non-speech sounds produced by the hands. The non-speech stimuli comprised auditory continua ranging from “clap” sounds (both hands clapped together) to “click” sounds (generated by striking the thumb on the middle finger).

The main aim of the current study was to further investigate the specificity of TMS-induced motor disruptions on auditory discrimination. We predicted that cTBS over the lip representation of M1 would impair discrimination of lip-articulated speech sounds (i.e., “ba” vs. “da”) but not of sounds that did not require the lips in their production. We also predicted that disruption of the lip motor representation would not affect discrimination of the non-speech control sounds produced by the hands. However, we anticipated a possible double-dissociation whereby cTBS over the hand motor representation would impair discrimination of non-speech but not speech sounds.

## MATERIALS AND METHODS

### PROCEDURE

Continuous TBS was applied over the left primary motor cortex at the level of either the lip or the hand representation. We assessed the behavioral effects of cTBS on the ability of participants to discriminate speech and non-speech sounds. The sound stimuli were drawn from three acoustic continua ranging between lip- and tongue-articulated phonemes (“ba”–“da”), another three continua created from phonemes that do not involve the lips in their articulation (“ga”–“da”) and three non-speech continua created from recordings of sounds made by the hands (“clap”–“click”).

**FIGURE 1:**
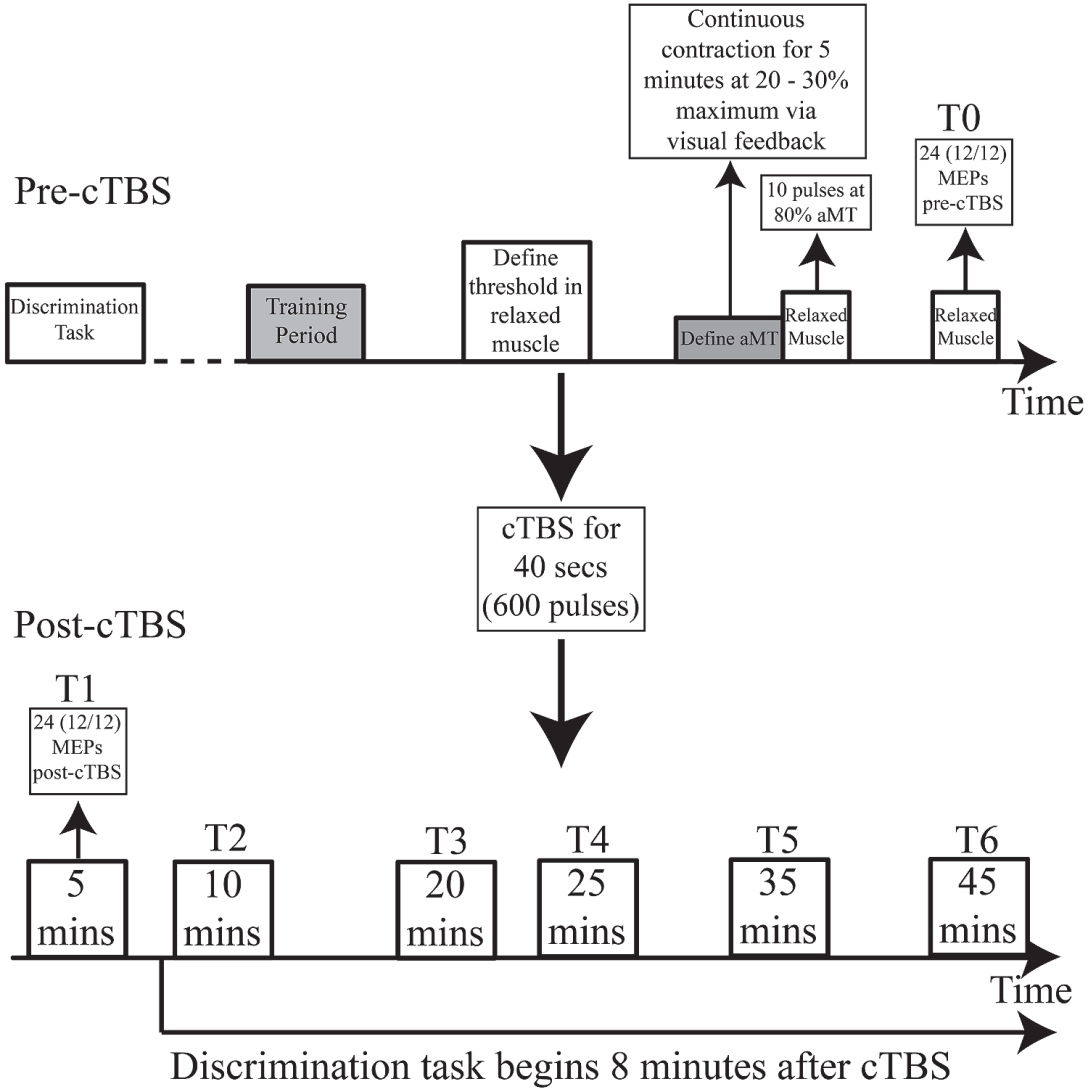
**Experimental procedure: cTBS applied for 40 s.** 12 MEPs recorded from the target (lip or hand) and non-target (lip or hand) muscle (24 in total) before cTBS (T0) and 5 (T1), 10 (T2), 20 (T3), 25 (T4), 35 (T5), and 45 (T6) min after cTBS. The pre-cTBS discrimination task was completed before TMS set-up began. The post-cTBS discrimination task began 8 min after theta-burst stimulation. aMT = active motor threshold.

Participants attended two testing sessions on separate days. During the first session, an identification task was carried out (see below for details). This allowed us to determine subject-specific logistic curves and category boundaries for the test continua prior to the second session. During the first session participants were also familiarized with the TMS equipment and set-up. This ensured that MEPs could be measured in both the contracted lip and hand muscles using single-pulse TMS. In the second session, participants performed an AXB-type discrimination task on the sound stimuli before and after receiving cTBS to either the hand or the lip representation. MEPs from the target muscle (lip or hand) were elicited using single pulse TMS to assess the effect of TBS on motor excitability (**Figure 1**).

### PARTICIPANTS

Twenty-seven right-handed native English speakers participated in this experiment. One participant withdrew due to discomfort during testing. Two participants did not complete the second session because they did not show categorical perception of either the speech or the non-speech sound continua in the first session. Data obtained from a fourth participant were excluded because the MEPs recorded during the second session were unreliable indicating a problem with the coil placement. Data from the remaining twenty-three participants were analyzed; hand stimulation group (*n =* 11, 18–45 years, 5 female), lip stimulation group (*n* = 12, 18–45 years, 5 female). All participants were medication-free with no personal or family history of seizures or other neurological disorders. All had normal or corrected-to-normal vision and reported no hearing problems. Informed consent was obtained from each participant before the experiment. All experiments were performed under permission from Oxfordshire NHS Research Ethics Committee B (REC Reference Number 10/H0605/7).

### BEHAVIORAL TASKS

Behavioral tasks were controlled and presented using Presentation software (Neurobehavioural systems) with all stimuli delivered through insert earphones (Etymotic Research). The earphones also served to protect the participant’s hearing during TMS. Participants were familiarized with all tasks and stimuli before testing.

#### Stimuli

Time-aligned averaging of periodic, aperiodic and F0 representations in the “Straight” channel-vocoder (Kawahara et al., 1999) was used to generate 10-step audio-morphed continua between pairs of naturally recorded speech (/ba/–/da/ and /ga/–/da/ speech syllables) and non-speech (/clap/–/click/) sounds (eight 10-step speech continua and four 10-step non-speech continua). To ensure that equivalent positions in the pairs of sounds were averaged, dynamic-time warping (www.ee.columbia.edu/~dpwe/resources/matlab/) was used implemented in Matlab (The Mathworks, Natick, MA, USA). This ensured that anchor points placed at evenly spaced positions in sound token 1 (at 50-ms intervals) could then be mapped onto a maximally similar corresponding position in sound token 2. This provides an automated means of creating high-quality, natural-sounding continua and allows us to use the proportion of sound token 1 compared to sound token 2 as a dependent measure when combining responses to different continua. For instance, for each pair, we generated 10 intermediate tokens as 10% acoustic steps from 5% (highly similar to sound 1, e.g., “ba” or “ga” or a “clap” sound) through to 95% (highly similar to sound 2, e.g., “da” or a “click” sound). A 45 or 55% sound token is likely to be heard as perceptually ambiguous, and may, for example, be interpreted as “ba” or “da” depending on the listener and the context.

#### Pilot experiment

The final stimuli were three “ba”–“da” continua produced by two male speakers and one female speaker, three “ga”–“da” continua spoken by one male speaker and two female speakers and three non-speech “clap”–“click” continua. These continua were chosen based on identification responses and category boundary values obtained from a pilot identification task. Sixteen native-English speaking participants (none of whom subsequently participated in the experiment described here) heard each of the 120 generated sound tokens (10 tokens for each of the 12 sound continua; eight 10-step speech continua and four 10-step non-speech continua) five times (600 tokens altogether split evenly across four blocks). They were then provided with a visual prompt 500 ms after stimulus offset highlighting two possible alternatives (e.g., “ba” or “da”) and responded with a key-press to indicate which of the two-alternatives they heard. A third-response alternative was also offered if participants believed they heard something other than the two-alternatives presented on the screen. Proportions of responses for each token were averaged over participants and transformed such that a logistic function could be fitted to the data for each pair and the position of the category boundary (i.e., the estimated morphing percentage for which equal numbers of sound token 1 and 2 responses might be expected) could be computed. Selecting the stimulus continua in this way ensured that (1) the category boundary was close to 50% for both speech and non-speech sounds and (2) that there was no significant difference in boundary position across the different stimulus continua [*F*(2,30) = 1.41, *p* > 0.1]. Analysis of occasions when listeners reported hearing something other than the two-alternatives presented on the screen revealed an average of 2.75% “other” responses (range 0.88–5.13%) for the nine chosen stimulus continua, (*F* < 1).

#### Identification task

In the identification task (first session), participants were presented with 12 repetitions of the 10 sound tokens from each of the nine continua (1080 trials in total split across four blocks) in a pseudo-randomized sequence. Participants saw a prompt 500 ms after stimulus offset indicating two possible alternatives (“ba”–“da”, “ga”–“da” or “clap”–“click”; left or right side stimulus presentation counterbalanced across trials) and responded with a key-press to indicate which of the two-alternatives they heard. Order of presentation of trials from each stimulus pair was pseudo-randomized across each of the blocks using MIX software (van Casteren and Davis, 2006). This ensured that no more than three exemplars from one stimulus pair (i.e., “clap”–“click”, “ba”–“da” or “ga”–“da”), no more than two exemplars from the same speaker and no more than two exemplars from the same point along the continuum were heard in succession. Interrogation of the subject-specific responses obtained during the identification task ensured that the category boundary position was between 35 and 65% for all participants and for all speech and non-speech continua.

#### Analysis of identification data

Logistic regression was used to fit curves to each participant’s identification data and obtain slopes (gradient) and the position of the category boundary for each acoustic continuum (i.e., “ba”–“da”, “ga”–“da” and “clap”–“click”). These were computed using the formula:

$$y=\frac{e^{\beta_{0}+\beta_{1}}}{1+e^{\beta_{0}+\beta_{1}}}$$

where *e* is the exponent function and β_0_+β_1_ refers to the regression line, with β_0_ representing the constant and β_1_ representing the gradient/regression coefficient. The higher the value of β_1_ the steeper the logistic curve (i.e., category boundary). By calculating the parameters of β_0_ (constant) and β_1_ (gradient) for this fitted function it is also possible to compute the position of the category boundary along the blended stimulus continuum, which corresponds to 50% accuracy on the y-axis.

#### Discrimination task

**FIGURE 2:**
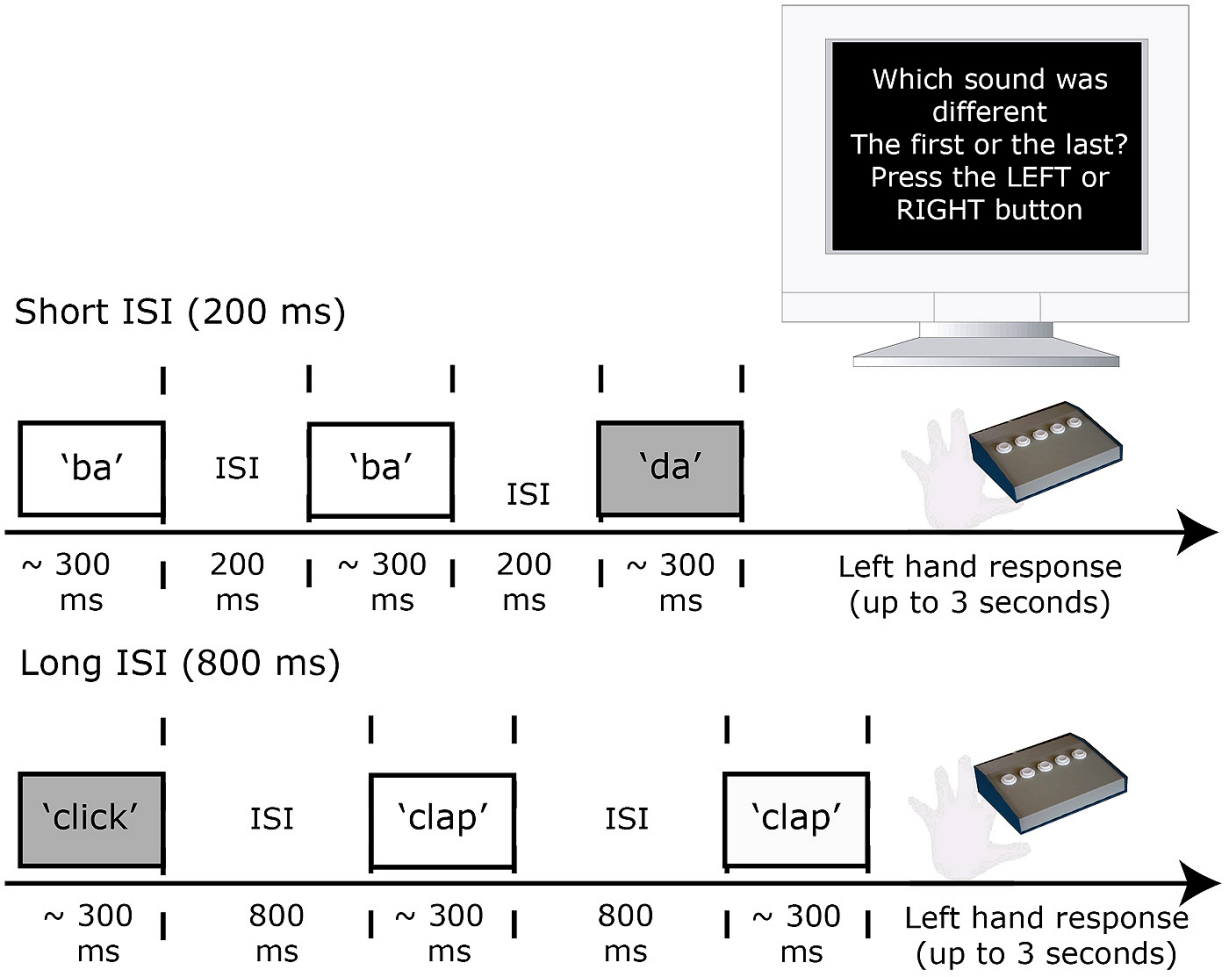
**Experimental design for the discrimination (AXB) task used pre and post stimulation.** The across- and within-category sound triplets were heard at two ISIs (200 and 800 ms). Responses were made via a button-press response with the left hand. After 3 s without response the next trial began.

In the discrimination task (second session), participants heard triplets of sounds one of which differed from the other two by three steps (i.e., 30%) along the acoustic continuum from which the sounds were drawn. The 30% change along the continuum could be either within-category (5% vs. 35% or 65% vs. 95%) or across-category (35% vs. 65%). Participants were required to indicate as accurately as possible with a left-hand button press whether the first (A) or the last sound (B) was different to the middle sound (X) in a triplet. An equal number of AAB and ABB type trials were presented with the stimulus pairs presented in both a forward (e.g., “ba”–“ba”–“da”, “ba”–“da”–“da”) and backward (e.g., “da”–“da”–“ba”, “da”–“ba”–“ba”) direction. This ensured that the position of the “different” sound in the triplet was not predictable. Thus there were three triplets (two within category and one across category) for each of the nine generated continua (three continua per contrast; three contrasts) that were presented as AAB or ABB, forwards and backwards (3 × 9 × 2 × 2 = 108 triplets). Each triplet was repeated three times with an inter-stimulus interval (ISI) between sounds in each triplet that was either 200 or 800 ms (six times in total; 648 stimuli; see **Figure 2**).

The AXB-type discrimination task was performed before and after cTBS. Before cTBS, the task was split into three blocks between which participants rested. Participants also received a 15-min break after completion of the discrimination task and prior to receiving cTBS. The post-rTMS discrimination task began 8-min after cTBS. At fixed time-points after cTBS, visual cues appeared on screen alerting the participant to “STOP. Take a break.” And the experimenter was cued to “Apply TMS now.” Single pulses of TMS were applied during the breaks over both the hand and the lip representation to elicit MEPs in the target and non-target muscles. All participants completed the discrimination task prior to the 45-min time-point at which the final set of 24 MEPs was recorded or by 50 min (shortly after the final set of 24 MEPs).

### ELECTROMYOGRAPHY (EMG)

Electromyography (EMG) activity was recorded from the lip and hand muscle site via surface electrodes (22 mm × 30 mm Kendall®; ABRO neonatal electrocardiogram electrodes). The electrodes were attached to the right corners of the upper and lower lip (orbicularis oris) and to the first dorsal interosseous (FDI) muscle and index finger of the right hand. The ground electrode was attached to the right temple in all cases. EMG signals were amplified, band-pass filtered (0.1–1000 Hz) and sampled (5000 Hz) via a CED 1902 four-channel amplifier, a CED 1401 analog-to-digital converter and a PC running Spike2 software (version 7; Cambridge Electronic Design).

All participants completed an initial “training period” during which they were required to produce a constant level of contraction of the lip or hand muscles via visual feedback indicating the level of online EMG activity displayed as power spectra. This training period continued for approximately 5 min or until a satisfactory level of 20–30% maximum voluntary contraction was reached as determined by two experimenters. Participants produced this level of contraction whilst single pulses of TMS were applied over the cortex to determine the active motor threshold (aMT) at the hand or lip representation “hot spot” (Möttönen et al., 2014).

### TMS

Magnetic stimulation was given over the hand or lip area of motor cortex and delivered using a hand-held 70 mm figure-eight coil (Magstim Co., Whitland, Camarthenshire, UK). Monophasic single pulses were generated by a Magstim 200 stimulator and used to elicit MEPs. Biphasic pulses were generated by a Magstim Super Rapid^2^ and used to define the aMT and deliver cTBS. The coil was placed tangentially to the scalp with the induced current flowing posterior–anterior under the junction of the two wings of the coil. The position and angle of the coil over the lateral surface was adjusted until a reliable MEP was observed in the targeted contralateral muscle (Möttönen et al., 2014). TMS was applied according to current safety guidelines (Wassermann, 1998; Rossi et al., 2009) with all participants required to complete a TMS safety screening form based on recommendations from Rossi et al. (2009). As there are no safety guidelines for the use of cTBS currently, the protocol of Huang et al. (2005) was followed.

#### Continuous theta-burst stimulation (cTBS)

Theta-burst TMS was applied continuously for 40 s over either the lip or hand representation of M1 cortex. The train of stimulation comprised 600 pulses, in high-frequency (50 Hz) triplets repeated at 5 Hz. The aMT for each participant was determined using the Magstim Super Rapid^2^ stimulator as the intensity at which single TMS pulses elicited more than 5 out of 10 MEPs with amplitude of at least 200 μV when the muscle was contracted at 20–30% of the maximum. The aMT was determined whilst participants maintained voluntary contraction of the hand or lip muscle at 20–30% of the maximum for 5 min. This was based on previous findings revealing that continuous contraction of the target muscle for 5 min influences the after-effects of cTBS (e.g., Gentner et al., 2008; Iezzi et al., 2008). Using the MagStim Super Rapid^2^, the average aMT (percentage of maximum stimulator output, ± SEM) for the lip area was 65.17% (±2.41%) and for the hand area was 51.55% (±2.15%). cTBS was delivered at an intensity of 80% aMT while participants relaxed their lip and hand muscles. This ensured that intensities used were sub-threshold and, therefore, not strong enough to elicit MEPs at rest. This was confirmed by administering 10 single-pulses of TMS at 80% aMT while the lip and hand muscles were relaxed. No MEPS were observed in participants at the intensity of stimulation used to apply cTBS. The maximum possible theta-burst intensity of 50% maximum stimulator output was applied if the intensity at 80% aMT was greater than 50%. The mean intensity used during cTBS over the lip area of M1 was 49.08% (±0.47%). The mean intensity used during cTBS over the hand area was 41.18% (±1.7%). Following cTBS, participants were told not to contract their lip or hand muscles until after the experiment had finished as activation during or following cTBS has previously been shown to alter the after-effects (Huang et al., 2008).

#### Single pulse TMS

To assess the suppressive effects of cTBS on cortical excitability, single-pulse TMS was used to elicit MEPs from the target (lip or hand) and non-target (lip or hand) muscle before cTBS and 5, 10, 20, 25, 35, and 45 min later for comparison with MEPs collected pre-cTBS. Twenty-four MEPs were acquired prior to cTBS and at each time point post-cTBS (12 MEPs per muscle) with an inter-pulse interval ranging from 5 to 6.5 s (*M* = 5.75, SD = 0.65). MEPs were acquired from the lip muscle first followed by the hand muscle in all cases. The intensity used to administer the single pulses of TMS in each participant was determined using a MagStim200 prior to the aMT described above. The intensity was defined as that which produced MEPs with average peak-to-peak amplitude of 0.3 mV or 1 mV on 10 consecutive trials for the lip and hand muscles, respectively (Möttönen and Watkins, 2009; Murakami et al., 2011; Möttönen et al., 2014). All MEPs before and after cTBS were recorded from the relaxed muscles. The average intensity (±SEM) used to elicit MEPs in the lip muscle was 64% (±1.59%) and 53% (±3.86%) in the hand muscle. We note that the stimulator outputs differ between the Magstim 200 that generates monophasic pulses and the Super Rapid2 that generates biphasic pulses. Therefore, the percentage (%) of stimulator output used to elicit MEPs in a relaxed muscle and for the aMT in a contracted muscle on each of these stimulators is not comparable.

### STATISTICAL ANALYSIS

For the behavioral data from the discrimination task, anticipation responses that were shorter than 200 ms were removed from the data (0.35% of total responses). If the participant did not respond within three seconds, then the next trial began (1.3% of responses were missed). Percent correct AXB responses for the across- and within-category stimuli were calculated for each contrast and each ISI separately. The scores post-cTBS were averaged across three time-bins; an early time bin (8–20 min post cTBS), a middle time-bin (20–35 min post cTBS) and a late time bin [35 min post cTBS–completion of the experiment (between 45 and 50 min)]. Missing data were replaced with the group mean for that contrast and ISI to allow the full ANOVA to be carried out (missing data occurred only at the late post-cTBS time point; 5/144 responses for the lip group and 12/132 responses for the hand group). For the two groups of participants who received lip (*n* = 12) and hand (*n* = 11) stimulation, two separate repeated-measures ANOVAs were carried out for the across- and within-category data. Within-subject effects of time (four levels: pre-cTBS, early, middle and late post-cTBS), ISI (200 vs. 800 ms), and stimulus type (three types: lip- and tongue-articulated and non-speech continua) were evaluated. *Post hoc* pairwise comparisons were used to compare time-points for the separate continua and ISI and were corrected using Bonferroni correction.

For the MEP data, MEPs with peak-to-peak amplitude greater than two SDs from the mean at each separate time point were removed as outliers (2.8% of responses). The remaining MEPs were averaged for the pre-cTBS time point, and the early post-cTBS (5 and 10 min), middle post-cTBS (20 and 25 min) and late post-cTBS (35 and 45 min) time points. A repeated-measures ANOVA with time (four levels: pre-cTBS, early, middle and late post-cTBS) as a within-subject factor was used to evaluate the effects of cTBS on motor cortex excitability for the lip and the hand data separately.

## RESULTS

### CATEGORICAL PERCEPTION OF SPEECH AND NON-SPEECH SOUNDS

**FIGURE 3:**
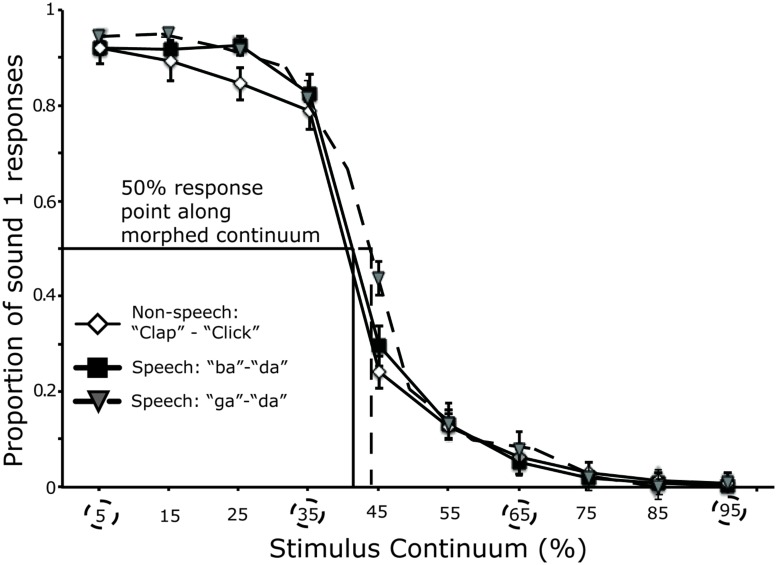
**Performance in the identification task (first session) across all participants.** A logistic curve was fit to the raw data points across participants. The circles depict the points along the continuum defined as within- or across-category. Lines on the *x-* axis mark the position of the category boundary for each stimulus pair. Error bars represent the SE of the mean.

Categorical perception of audio-morphed speech and non-speech continua averaged across all participants tested in session 1 (*n* = 23) is shown in **Figure 3**. The category boundary position for all stimulus continua in all participants was between the 35% and 65% along the acoustic continuum. Analysis of the slopes (β_1_) across stimulus continua revealed no significant difference in the steepness of the logistic curves (*F* < 1; “ba”–“da” = 0.90 ± 0.02; “ga”–“da” = 0.90 ± 0.02; “clap”–“click” = 0.92 ± 0.02). There was no significant difference in boundary position across stimulus pair (*F* < 1), (“ba”–“da”: mean = 43.12 ± 1.18% da; “ga”–“da”: mean = 44.39 ± 1.07% da; “clap”–“click”: mean = 42.33 ± 1.01% click).

**FIGURE 4:**
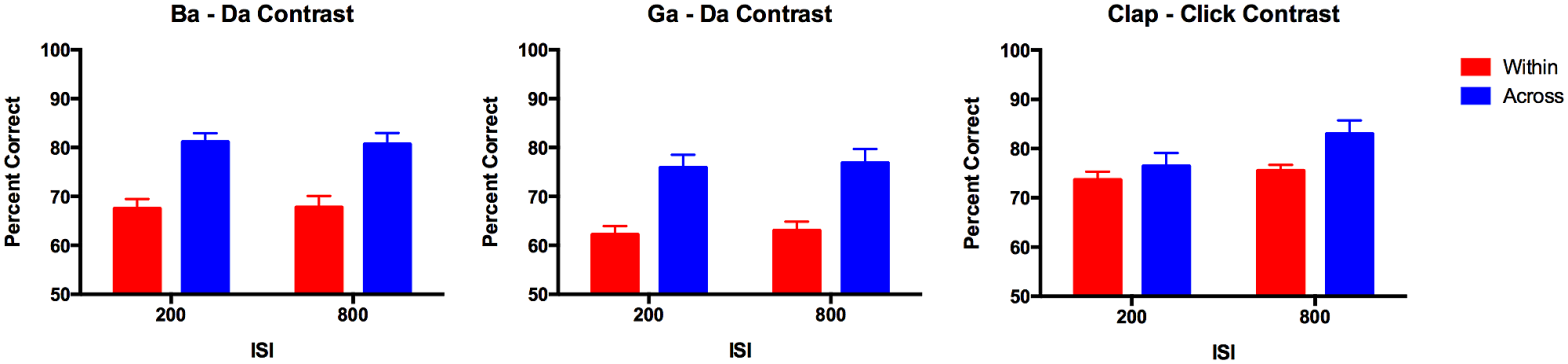
**Pre-stimulation discrimination accuracy for across and within-category stimuli.** Bars represent the group mean (*n* = 23); error bars represent the SE of the mean. The three contrasts each showed the typical pattern associated with categorical perception wherein across category pairs are discriminated more robustly than within-category pairs. This pattern is weakest for the non-speech contrast but was still significant.

The data from the discrimination task that was performed before cTBS was combined for both groups of participants (*n* = 23) and analyzed using ANOVA with within-subjects factors of contrast (three types: ba–da, ga–da, and click–clap), stimulus type (across vs. within category) and ISI (200 vs. 800 ms); the between-subject factor of group was included but was not expected to be a main effect or interact significantly with any of the other factors. As expected for stimuli that are perceived categorically, accuracy on discrimination of across-category stimuli was significantly better than for within-category stimuli that had an equivalent acoustic difference (i.e., 30%) between them [*F*(1,22) = 83.30, *p* < 0.0005]. This main effect of stimulus type interacted significantly with the contrast, however [*F*(2,42) = 4.83, *p* = 0.013]; the main effect of contrast was significant also [*F*(2,42) = 17.27, *p* < 0.0005] due to significantly lower performance on the ga–da contrast compared with the other two contrasts (ba–da, *p* = 0.002, clap–click, *p* < 0.0005, corrected). The interaction between stimulus type and contrast was explored with separate ANOVAs for within and across category stimuli. This revealed a significant difference among the three contrasts for the scores on the within-category stimuli [*F*(2,42) = 21.24, *p* < 0.0005] but no difference among the scores for the across-category stimuli. The within-category stimuli were discriminated significantly more accurately for the non-speech clap–click contrast relative to the other two contrasts (ba–da: mean difference = 6.96 ± 2.00%, *p* = 0.007; ga–da: mean difference = 11.95 ± 1.85%, *p* < 0.0005); the within-category stimuli were also discriminated more accurately for the ba–da contrast relative to the ga–da contrast (mean difference = 5.00 ± 1.66%, *p* = 0.020; corrected; see **Figure 4**). The non-speech contrast was also the only one to show a significant difference in accuracy according to ISI [*F*(1,22) = 5.58, *p* = 0.027]; performance on the longer ISI was better than for the shorter ISI. The interaction between ISI and stimulus type was not significant, however.

In sum, all three types of contrast (ba–da, ga–da, and clap–click) were perceived categorically before cTBS was applied to either the hand or the lip representation in M1. Although there were no significant differences among the slopes of the identification functions for the three different contrasts, the better performance on the within-category discrimination of the clap–click contrast relative to the other two contrasts suggests that the non-speech contrast was perceived less categorically than the speech contrasts (see **Figure 4**).

### THE EFFECT OF cTBS ON DISCRIMINATION OF SPEECH AND NON-SPEECH SOUNDS

#### Discrimination of across-category stimuli

**FIGURE 5:**
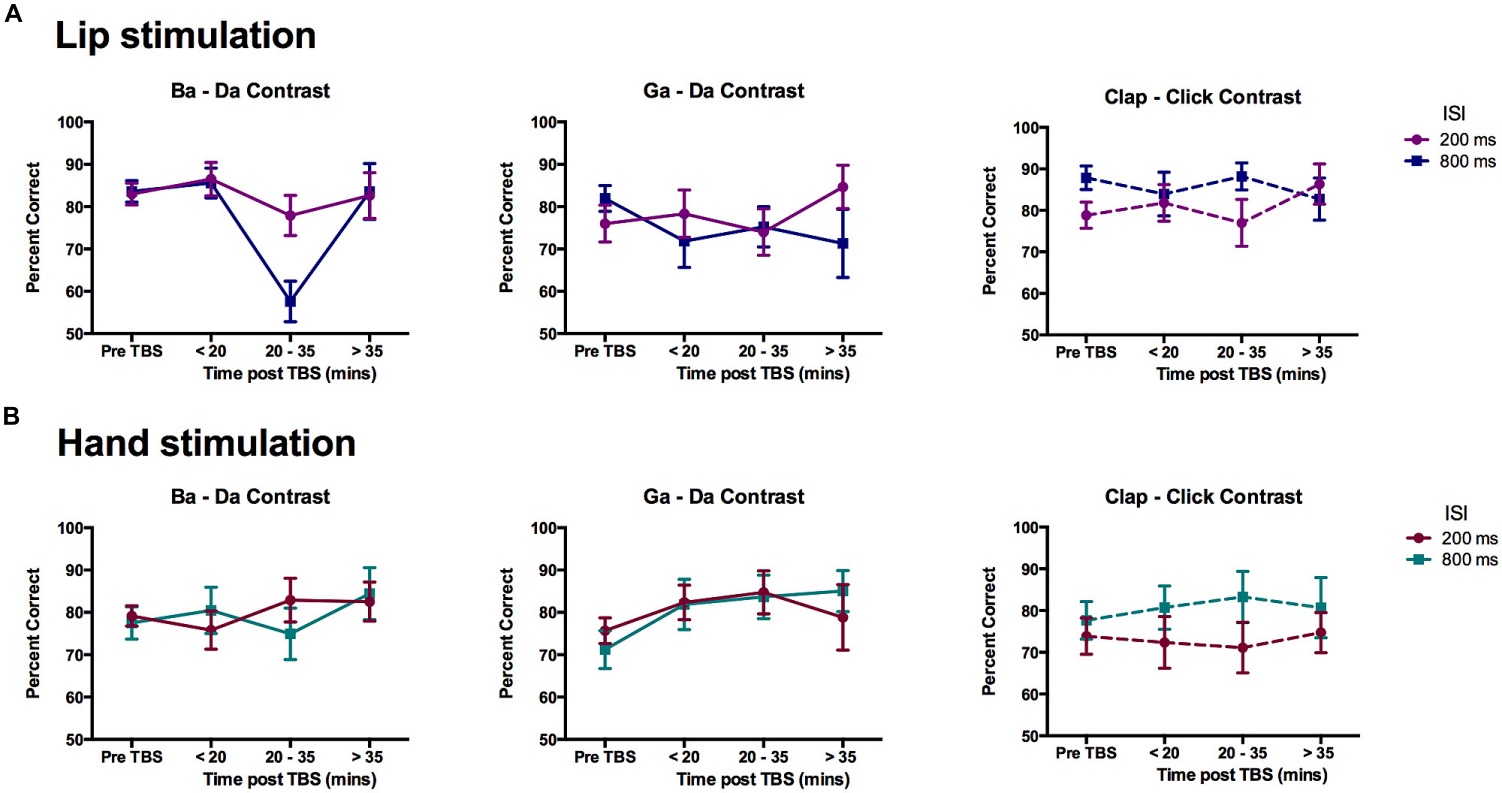
**Effects of cTBS on discrimination of across-category pairs.** Mean percent correct scores for the participants in the **(A)** Lip stimulation group (*n* = 12) and **(B)** Hand stimulation group (*n* = 11). Data are plotted separately for each contrast. The graphs show the data for each time point in the experiment with the two ISI plotted as separate lines. Error bars represent the SE of the mean. The only significant reduction in performance was seen for the data obtained between 20 and 35 min post-cTBS to the lip representation for the lip-articulated “ba”–“da” contrast.

Change in discrimination accuracy due to cTBS for across-category stimuli was evaluated using ANOVA with within-subject factors of contrast (three types: ba–da, ga–da, clap–click), time (four time-points: pre-cTBS, early, middle, and late post-cTBS), and ISI (200 vs. 800 ms). For the group of participants that received cTBS over the lip representation (*n* = 12), there was a close to significant three-way interaction following a Greenhouse–Geisser correction to the degrees of freedom because of non-sphericity of data (Mauchly’s test of sphericity, *p* = 0.036; *F*(3.46,38.03) = 2.258, *p* = 0.089). The two-way interaction between contrast and time was significant [*F*(6,66) = 2.355, *p* = 0.040] as was the main effect of time [*F*(3,33) = 6.144, *p* = 0.002]. Separate ANOVAs for the three different contrasts revealed that the two-way interaction between contrast and time was due to a significant effect of time for the “ba”–“da” speech contrast [*F*(3,33) = 9.291, *p* < 0.0005] and not for the other two contrasts (ga–da and clap–click). *Post hoc* pairwise comparisons revealed the main effect of time in the “ba”–“da” contrast was due to a significant reduction in performance at the middle post-cTBS time point relative to all others (mean difference ± SEM for middle post-cTBS compared with: pre-cTBS = 15.54 ± 2.83%, *p* = 0.001; early post-cTBS = 18.29 ± 3.98%, *p* = 0.005; late post-cTBS = 15.34 ± 4.07%, *p* = 0.019; *p*-values corrected). This effect was greater for triplets presented with ISI of 800 ms than for those with ISI of 200 ms at the middle time point [*t*(11) = 3.18, *p* = 0.009; **Figure 5A**]. Note, however, that the time by ISI interaction for the “ba”–“da” contrast did not quite meet the *p* < 0.05 cutoff for significance [*F*(3,33) = 2.748, *p* = 0.058]. One-sample *t*-tests were also used to test if discrimination of “ba”–“da” stimuli was above chance (50%) at the middle time point. Only the discrimination of “ba”–“da” stimuli presented with a short ISI (200 ms) was significantly above chance [*t*(11) = 5.90, *p* < 0.0005]; discrimination of triplets presented with a longer ISI (800 ms) did not differ from chance performance (*p* = 0.140).

For the group of participants that received cTBS over the hand representation (*n* = 11), there were no significant main effects or interactions (**Figure 5B**); there was a close-to-significant interaction between contrast and ISI [*F*(2,20) = 3.40, *p* = 0.054], which appears to be due to better performance at the longer ISI for the non-speech (“clap”–“click”) contrast across all time-points (see also results above for the pre-TBS data analysis).

#### Discrimination of within-category stimuli

**FIGURE 6:**
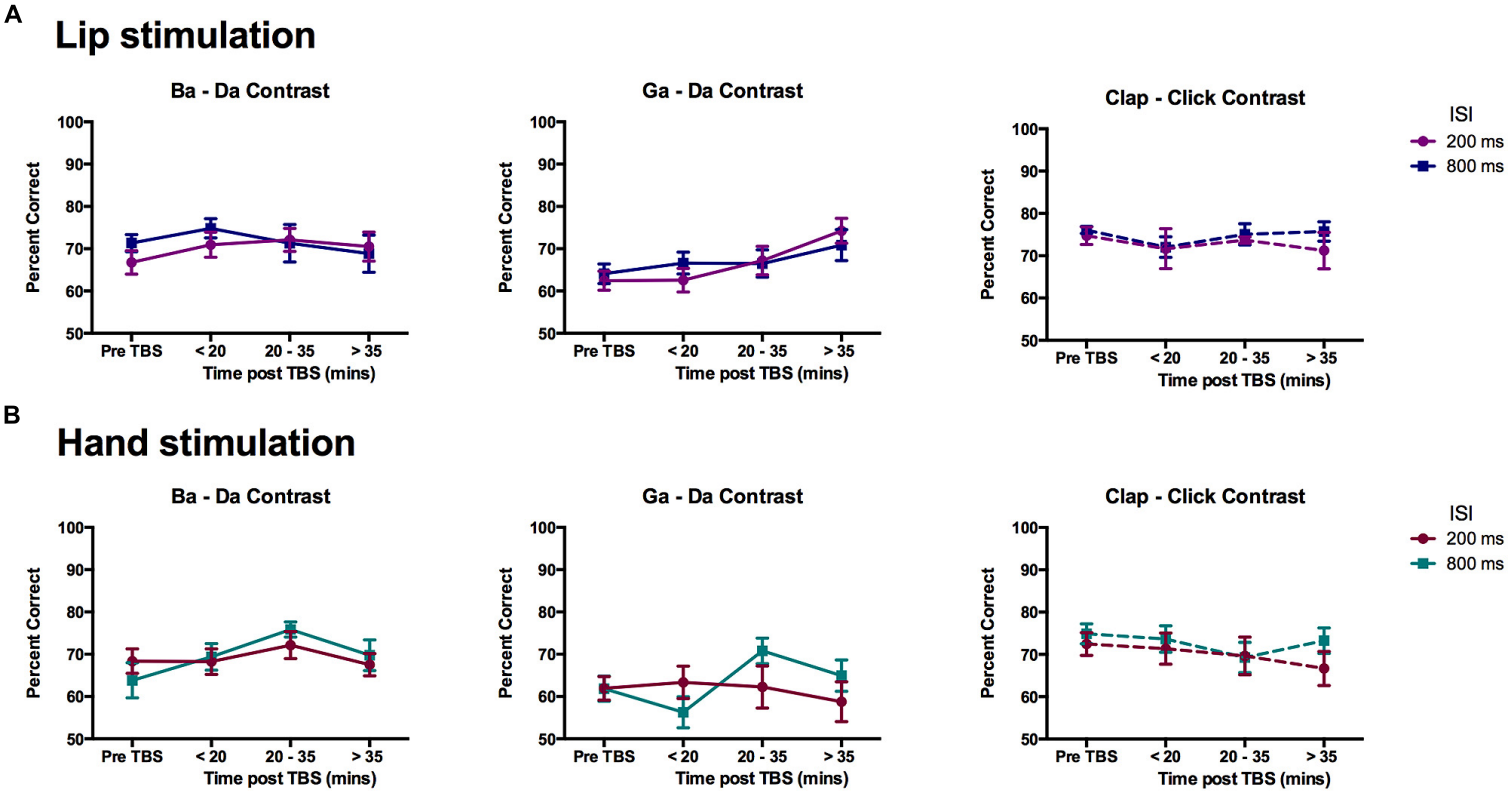
**Effects of cTBS on discrimination of within-category pairs.** Mean percent correct scores for the participants in the **(A)** Lip stimulation group (*n* = 12) and **(B)** Hand stimulation group (*n* = 11). A significant improvement in discrimination accuracy for the “ga”–“da” contrast in the group who received cTBS to the lip representation can be seen in the middle graph of the top row. In the hand stimulation group, the performance on the “ga”–“da” contrast was significantly lower than that for the non-speech contrast.

Change in discrimination accuracy due to cTBS for within-category stimuli was evaluated using ANOVA as described above with within-subject factors of contrast (three types: ba–da, ga–da, clap–click), time (four time-points: pre-cTBS, early, middle, and late post-cTBS), and ISI (200 vs. 800 ms). For the group of participants that received cTBS over the lip representation (*n* = 12), there was a significant interaction between contrast and time [*F*(6,66) = 2.37, *p* = 0.039] and a significant main effect of contrast [*F*(2,22) = 10.50, *p* = 0.001]. The main effect of contrast was due to lower performance on the within-category discrimination for the “ga”–“da” contrast compared to the non-speech “clap”–“click” contrast (mean difference = 6.97 ± 1.37%, *p* = 0.001), which suggests a more typical categorical perception performance for the speech compared to the non-speech stimuli (see results above for the pre-TBS data analysis). Separate ANOVAs for each of the three different contrasts showed a significant main effect of time [*F*(3,33) = 6.83, *p* = 0.001] for the “ga”–“da” contrast but not for the other speech (“ba”–“da”) nor the non-speech (“clap”–“click”) contrasts. The discrimination of within-category stimuli was significantly better at the late post-cTBS time point compared to the pre-cTBS time point (mean difference = 9.29 ± 1.99%, *p* = 0.004, corrected) and the early post-cTBS time point (mean difference = 7.97 ± 2.38%, *p* = 0.039, corrected), indicating improved performance over the course of the experiment in discriminating within-category stimuli for the “ga”–“da” speech contrast (see **Figure 6A**). For the group of participants that received cTBS over the hand representation (*n* = 11), there was a significant main effect of contrast [*F*(2,20) = 12.22, *p* < 0.0005]; none of the other main effects or interactions were significant, though the main effect of ISI was close [*F*(1,10) = 4.01, *p* = 0.073; **Figure 6B**].

#### Summary of discrimination results

In sum, cTBS over the lip but not the hand representation in M1 significantly reduced the ability of participants to discriminate speech sounds that are lip articulated from those that are tongue articulated but not their ability to discriminate speech sounds from different phonetic categories that are both tongue articulated nor non-speech sounds made by the hands. The reduction in discrimination ability was observed only at a time point occurring 20 min after the stimulation and was not seen in the data obtained earlier at 5 and 10 min post stimulation or later at 35 and 45 min post stimulation. Discrimination of the lip-articulated speech sounds dropped to chance level at this middle time-point when they were presented with an ISI of 800 ms, whereas discrimination performance for stimuli presented with an ISI of 200 ms was slightly reduced but remained significantly above chance.

### THE EFFECT OF cTBS ON MOTOR EXCITABILITY

**FIGURE 7:**
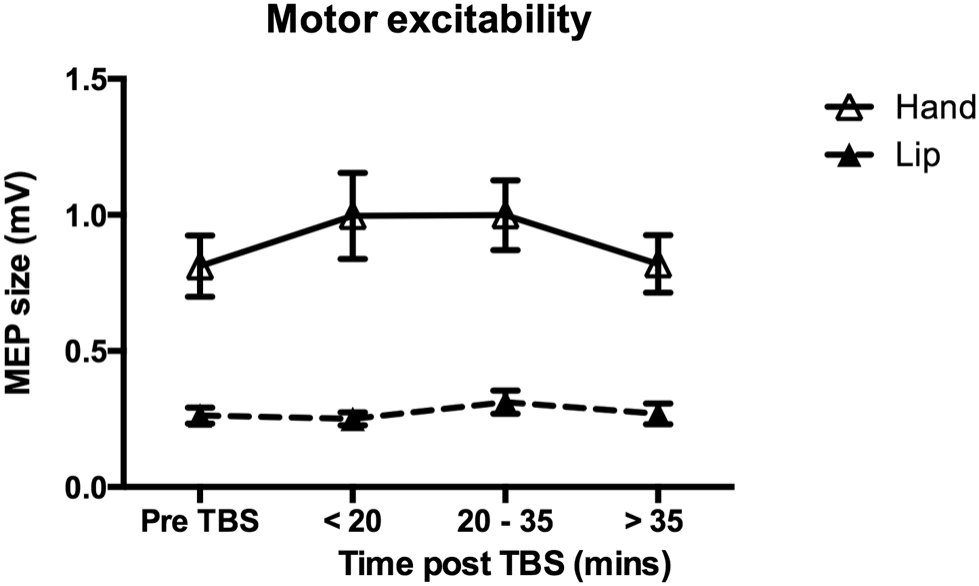
**Effect of cTBS on motor excitability.** Group mean MEP sizes at each time point (pre-cTBS, early, middle and late post-cTBS) are shown for the target muscle in response to single-pulse TMS over either the Lip (dashed line) or the Hand (solid line) representation in M1. Error bars represent SEs of the mean. There was no significant change in excitability due to cTBS in either the Lip (*n* = 12) or the Hand (*n* = 11) group. The difference in amplitudes for the MEPs elicited in the Lip compared to the Hand muscle was expected as this was used in determining the thresholds separately for each muscle (see Section “Materials and Methods” for details).

For the data obtained from the lip target muscle, there was no significant effect of cTBS on MEP size [*F*(3,33) = 1.34, *p* = 0.277]. Similarly, cTBS over the hand representation, had no significant effect on MEP size recorded from the hand target muscle [*F*(3,30) = 2.11, *p* = 0.120; **Figure 7**]. Analysis of MEPs recorded from the non-target muscle also revealed no significant change in motor excitability, *F* < 1. In sum, for the group data, 40-s cTBS over either the lip or hand representation in M1 did not significantly change motor excitability in either area as indexed by the size of MEPs elicited by single pulse TMS. Nevertheless, we wished to explore whether the reduction in discrimination ability seen at the time point occurring 20 min after the stimulation was related to the efficacy of cTBS to reduce MEP size in some of the participants. Five participants showed a decrease in MEP size at the middle time point relative to the pre-cTBS MEP size (3.5–16% reduction) and seven participants did not. Performance of these two subgroups on the discrimination of the “ba”–“da” speech contrast at the two ISIs was compared using independent *t*-tests. There were no significant differences. However, the subgroup showing decreased motor excitability had a lower mean performance (49.8%; i.e., chance) on discrimination of the stimuli at the longer ISI compared to the subgroup that did not show a reduction in MEP size (63.2%).

## DISCUSSION

To our knowledge, this is the first study to assess the effects of cTBS on discrimination of naturally recorded speech and non-speech sounds. By applying 40 s of cTBS over the lip representation in the motor cortex, we temporarily impaired the ability of listeners to discriminate syllables from different phonetic categories on a continuum that varied in place of articulation from the lips to the tongue (“ba” to “da”); the ability to discriminate syllables from within a phonetic category was unaffected by cTBS. The impairment observed was maximal between 20 and 35 min after the stimulation for the across-category “ba”–“da” stimuli presented with a longer ISI (800 ms) and, in fact, discrimination performance at this time was reduced to chance levels. Discrimination of the same stimuli presented with a short ISI (200 ms) at the same time-point was slightly less affected by the cTBS and remained above chance. The finding that the impairment occurred during a 15-min time period starting 20 min after cTBS was applied is consistent with the time period at which the maximum inhibitory effects of cTBS on motor excitability have been previously reported (Huang et al., 2005). Data obtained earlier (i.e., in the first 20 min following stimulation) and later (i.e., more than 35 min after the stimulation) did not show any changes in discrimination ability relative to the pre-stimulation baseline data. This impairment in discrimination of speech sounds was not seen when the hand representation of the motor cortex was stimulated. These results support previous studies that have shown a mediating role of the motor system in speech perception with performance in speech tasks significantly affected following TMS over the motor regions (e.g., Meister et al., 2007; D’Ausilio et al., 2009; Sato et al., 2009; Bartoli et al., 2013). These findings also replicate our previous results using low frequency (0.6 Hz) repetitive TMS for 15 min to temporarily disrupt function in the lip representation of primary motor cortex, impairing categorical perception and discrimination of speech syllables that involved the lips in their production (Möttönen and Watkins, 2009). There were a number of important differences between the two studies, however, and these are discussed below.

Firstly, 40-s of cTBS was used in the current study because we anticipated that this would induce a longer-lasting disruptive effect than that induced by 15 min of low frequency repetitive TMS, which we used previously. We found that the short train of high-frequency stimulation was well tolerated by participants and the behavioral disruption lasted for about 15 min occurring 20 min after the train had ended. The timing and duration of the behavioral effect requires replication but the technique in general offers some useful potential applications in future studies on the neural basis of speech processing. For example, TBS has been used successfully in combination with paired pulse TMS to study connectivity during speech processing (Murakami et al., 2012). It might also be combined usefully with neuroimaging techniques to further investigate auditory-motor processing of speech sounds (see Möttönen et al., 2013, 2014).

Another important difference from our previous study is that in the current study we used natural speech sounds recorded from three different speakers and audio-morphed into continua using the “Straight” channel-vocoder (Kawahara et al., 1999). Previously, we used computer-generated artificial speech syllables and created a single continuum for each contrast by changing the slope of the formant transitions. Also, in the current experiment we included a novel non-speech contrast, creating three continua based on sounds made by the hands (“clapping”) and fingers (“clicking”), which were also perceived categorically. Including these stimuli allowed us to test for a possible double dissociation, whereby stimulation over the hand area might disrupt categorical perception of sounds made with the hands and not speech sounds whilst stimulation over the lip area might disrupt categorical perception of sounds made with the lips and not those made with the hands. Unfortunately, our findings are consistent with a single dissociation only, namely that the lip stimulation affected perception of speech sounds that were lip articulated and had no effect on the perception of sounds made by the hands; the hand stimulation did not impair discrimination of any auditory stimuli.

In the current study, the behavioral testing implemented used an AXB-type discrimination design with all stimulus sounds presented in a random order mixing the contrasts and continua tested. These included two different speech continua each from three speakers and three non-speech continua. Using the AXB discrimination task addresses criticism of our previous findings using low frequency rTMS and a same-different paradigm (Möttönen and Watkins, 2009) relating to the possibility that response bias changed rather than speech perception (Hickok, 2010). The previous study did not include identical pairs in the same–different task, which meant we could not evaluate changes in response bias using signal detection theory. The impaired discrimination of speech sounds reported in the current study cannot be explained by a change in response bias lending further support to our original claim that stimulation of the motor cortex impairs speech perception.

A final difference between the two studies was that we tested two different ISIs in the current experiment (200 vs. 800 ms), whereas we used 500 ms between stimulus pairs in our previous same-different task. Discrimination accuracy of lip- (“ba”) versus tongue-articulated (“da”) speech syllables was reduced to chance level 20 min after the cTBS over the lip representation only when the sound stimuli were presented at the longer ISI of 800 ms. Note, however, that the three-way interaction between contrast, time, and ISI was not quite significant and that discrimination of the same stimuli presented at this time-point with 200 ms ISI was slightly affected by cTBS also. This is a novel finding and is consistent with evidence suggesting that once auditory memory has faded listeners must rely on pre-established phonetic representations to distinguish between speech sounds (Pisoni, 1973; Massaro and Cohen, 1983; Gerrits and Schouten, 2004). We propose this difference reflects phonetic vs. acoustic perception; at the shorter ISI, participants are more reliant on auditory “echoic” memory whilst at the longer ISI the auditory information is lost and participants are reliant on pre-established phonetic categories. It is this ability that is impaired when discriminating between lip- (“ba”) versus tongue-articulated (“da”) speech sounds following cTBS over the lip motor representation. This is consistent with studies assessing the role of verbal working memory and articulatory rehearsal in phonological discrimination (Boatman, 2004; Gough et al., 2005; Romero et al., 2006; Sato et al., 2009). Compared to sham stimulation, rTMS applied over left ventral premotor cortex significantly disrupted the ability to perform phoneme discrimination (Romero et al., 2006) and phonemic segmentation (Sato et al., 2009). One interpretation is that rTMS temporarily disrupts the recruitment of articulatory-based motor representations during phonological processing that are dependent on phonemic segmentation cues and a phonological short-term working memory store (Baddeley, 1990; Zatorre et al., 1992; Burton et al., 2000).

We report no effect of cTBS over the lip motor representation on discrimination accuracy for sounds that do not require the lips for articulation (“da” vs. “ga”) and for non-speech sounds (“clap” vs. “click”). This shows that the impairment was specific to the articulatory features of the speech sounds. cTBS over the hand motor representation also had no effect on discrimination accuracy of speech or non-speech sounds showing that the temporary inhibition was specific to the lip rather than the hand motor representation. These results support previous studies investigating the contribution of articulatory motor cortex to perceptual speech processing and are consistent with claims that the lip motor representation contributes to speech perception in an articulator-specific manner (e.g., Möttönen and Watkins, 2009).

We also investigated the effects of applying 40 s of cTBS over the lip and hand representation of M1 on motor excitability, with 40 s of continuous stimulation shown to be more robust in inducing an inhibitory effect than protocols using 20 s of cTBS (e.g., Gentner et al., 2008). We found no significant inhibitory or facilitatory effect of cTBS over the lip or hand motor representation on MEPs recorded from the lip or hand target muscle. Thus, we did not replicate findings from previous studies revealing an inhibitory effect of 40 s of cTBS on motor excitability (e.g., Huang et al., 2005, 2008; Gentner et al., 2008). One possible account for why no effect of cTBS on the size of MEPs was observed in our study is that we recorded MEPs alongside the discrimination responses. Whilst all behavioral responses were made with the left hand, ipsilateral to the site of stimulation to avoid motor excitability changes due to hand movements, inter-hemispheric inhibition from the right motor cortex cannot be ruled out as affecting the left motor cortex excitability in an unexpected way. Increased attentional demands present during discrimination of the speech and non-speech sounds may also have contributed to the absence of an effect of cTBS on motor excitability.

A more likely explanation of our failure to replicate previous findings of reduced motor excitability following cTBS relates to recent reports of highly variable responses to cTBS across protocols and across participants. For example, applying cTBS for 20 s over left M1 facilitated rather than suppressed the amplitude of MEPs recorded from the contralateral hand (Gentner et al., 2008). Suppressed motor excitability occurred only when voluntary muscle contraction was performed before cTBS. By doubling the duration of stimulation (applying cTBS for 80 s instead of 40 s). Gamboa et al. (2010) found a reversed facilitatory rather than inhibitory effect showing that the latter is not increased by simply prolonging the period of stimulation. Recently, Hamada et al. (2013) also failed to replicate the suppression of motor excitability in a large group of healthy volunteers. They reported high inter-individual variability, which has been attributed to potential differences among individuals in the excitability of populations of neurons activated following cTBS (Day et al., 1987; Rothwell, 1997; Ridding and Ziemann, 2010; Hamada et al., 2013). A number of potential factors have been suggested that contribute to this variability including age, gender, time of day, hormonal influence (e.g., changes in cortisol levels), neuromodulators and genetics (Ridding and Ziemann, 2010). A systematic investigation of inter-individual variability for theta-burst protocols reported no consistent pattern of response among individuals related to age, gender, time of day or initial differences in stimulation intensity thresholds and baseline MEP amplitude (Hamada et al., 2013). Rather, Hamada et al. (2013) suggested that the inter-individual variation observed reflects differences between people in the population of neurons activated by theta-burst stimulation that might be determined by differences in cortical anatomy.

In the current study, we examined whether individuals who showed a reduction in motor excitability (as indexed by MEP amplitude changes) also showed a greater behavioral impairment. There was a trend in the data to support this view but the two subgroups of “responders” (*n* = 5) and “non-responders” (*n* = 7) were not significantly different in their ability to discriminate stimuli at the middle post-cTBS time-point when as a group they showed a significant decrement in task performance. Taking into account our own experience and the confusion in the literature, it is possible that MEPs are not always reliable indicators of the efficacy of cTBS on motor excitability.

## CONCLUSION

Using cTBS, we replicated our previous findings that temporary disruption of the lip motor representation impairs the perception of speech sounds that rely on the lips for their production. This impairment is not explained by a change in response bias as it was obtained using an AXB discrimination task. Furthermore, we found that the effect of the TMS-induced disruption occurs predominantly for discrimination that relies on pre-existing phonetic categories and affects discrimination that relies on shorter-term acoustic representations to a lesser extent. This novel finding arose from a longer behavioral testing session with a larger number of natural speech and non-speech continua that was afforded by the anticipated longer-lasting effects of cTBS relative to low-frequency rTMS. A further advantage of TBS is that this longer-lasting effect is brought about by a very brief stimulation train (40 s compared to 15 min of low frequency rTMS). The use of TBS for further studies of speech processing holds promise, therefore. The effect of cTBS on motor excitability in our study was negligible, however. Although this failure to replicate previous effects was unexpected, the literature supports a picture of high inter-individual variability in motor excitability changes in response to TBS. It is as yet unknown whether similar variability affects behavioral responses. Our findings suggest, however, that cTBS over the motor cortex can affect behavior even when changes in motor excitability are not reliable.

## Conflict of Interest Statement

The authors declare that the research was conducted in the absence of any commercial or financial relationships that could be construed as a potential conflict of interest.
